# Sarcopenia is linked to higher levels of B-type natriuretic peptide and its N-terminal fragment in heart failure: a systematic review and meta-analysis

**DOI:** 10.1007/s41999-024-00950-x

**Published:** 2024-03-08

**Authors:** Konstantinos Prokopidis, Jordi Morwani-Mangnani, Garry McDowell, Gregory Y. H. Lip, Massimo Venturelli, Rajiv Sankaranarayanan, Masoud Isanejad

**Affiliations:** 1 Department of Musculoskeletal Ageing and Science, Institute of Life Course and Medical Sciences, University of Liverpool, Liverpool, UK; 2 Leiden, The Netherlands; 3 Liverpool Centre for Cardiovascular Science at University of Liverpool, Liverpool John Moores University and Liverpool Heart and Chest Hospital, Liverpool, UK; 4 School of Pharmacy and Biomolecular Sciences, Liverpool John Moores University, Liverpool, UK; 5 Research Lab, Liverpool Heart and Chest Hospital, Liverpool, UK; 6 Danish Center for Clinical Health Services Research, Department of Clinical Medicine, Aalborg University, Aalborg, Denmark; 7 Department of Neurosciences, Biomedicine and Movement Sciences, University of Verona, Verona, Italy; 8 Liverpool University Hospitals NHS Foundation Trust, Liverpool, UK

**Keywords:** Heart failure, Sarcopenia, BNP, NT-proBNP, Biomarkers, Cardiac function

## Abstract

**Aims:**

Sarcopenia is linked to impaired physical function and exercise tolerance. The aim of this systematic review and meta-analysis was to examine the association of sarcopenia and low appendicular skeletal muscle (ASM) with biomarkers of cardiac function, B-type natriuretic peptide (BNP) and its N-terminal fragment (NT-proBNP), in patients with heart failure (HF).

**Methods and results:**

From inception until May 2023, a systematic literature search of observational studies was undertaken utilizing the PubMed, Web of Science, Scopus, and Cochrane Library databases. A meta-analysis employing a random-effects model was used to compute the pooled effects (CRD42023418465). Overall, 16 studies were included in this systematic review and meta-analysis. Our main analysis showed that sarcopenia in HF was linked to significantly higher levels of BNP (MD: 87.76, 95% CI 20.74–154.78, *I*^2^ = 61%, *P* = 0.01) and NT-proBNP (MD: 947.45, 95% CI 98.97–1795.93, *I*^2^ = 35%, *P* = 0.03). Similarly, low ASM was associated with significantly higher levels of BNP (MD: 118.95, 95% CI 46.91–191.00, *I*^2^ = 93%, *P* < 0.01) and NT-proBNP (MD: 672.01, 95% CI 383.72–960.30, *I*^2^ = 2%, *P* < 0.01). The quality of the included cohort studies was considered moderate, using the binary AXIS checklist and the Cochrane Tool to Assess the Risk of Bias in Cohort Studies.

**Conclusions:**

In patients with HF, sarcopenia and reduced ASM are associated with considerably higher plasma levels of BNP and NT-proBNP. Future research is required to investigate whether sarcopenia may express dysregulated biomarkers of cardiac function.

**Graphical abstract:**

**Supplementary Information:**

The online version contains supplementary material available at 10.1007/s41999-024-00950-x.

## Key summary points

**Aim:**

To evaluate the association of sarcopenia and low appendicular skeletal muscle mass with B-type natriuretic peptide (BNP) and its N-terminal fragment (NT-proBNP) levels in patients with heart failure.

**Findings:**

Sarcopenia was associated with significantly greater levels of BNP (MD: 87.76, 95% CI 20.74 – 154.78, *I*^2^ = 61%, *P* = 0.01) and NT-proBNP (MD: 947.45, 95% CI 98.97 – 1795.93, *I*^2^ = 35%, *P* = 0.03). Likewise, low appendicular skeletal muscle mass was linked to higher levels of BNP (MD: 118.95, 95% CI 46.91 – 191.00, *I*^2^ = 93%, *P* < 0.01) and NT-proBNP (MD: 672.01, 95% CI 383.72 – 960.30, *I*^2^ = 2%, *P* < 0.01).

**Message:**

Research is needed to determine whether sarcopenia may be a contributor to dysregulated plasma concentrations of natriuretic peptides.

**Supplementary Information:**

The online version contains supplementary material available at 10.1007/s41999-024-00950-x.

## Graphical abstract

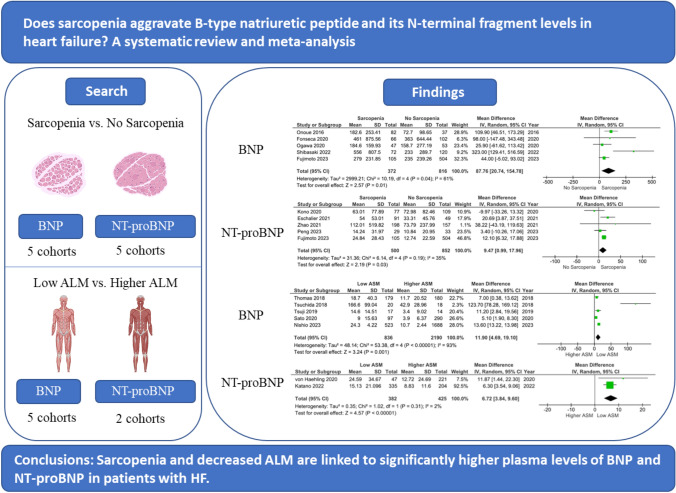

## Introduction

Heart failure (HF) and sarcopenia are two common age-related conditions that often coexist in older adults [1]. Sarcopenia is defined as the loss of muscle mass, strength, and function, and is known to be associated with a variety of adverse health outcomes [2]. HF, on the other hand, is a clinical syndrome that results from structural or functional impairments of the heart and is characterized by multiple symptoms, such as breathlessness, fatigue, and fluid retention [3].

There is growing evidence to suggest that the presence of HF may accelerate the development of sarcopenia [4]. Epidemiological studies conducted in older adults have consistently shown that individuals with HF have a higher prevalence of sarcopenia compared to those without HF [5, 6]. In addition, individuals with HF tend to have greater declines in muscle mass and strength over time, which further contributes to the progression of sarcopenia [7, 8].

Nevertheless, the question of whether the presence of sarcopenia during HF accelerates HF progression remains unclear. Some studies have suggested that sarcopenia may be an independent predictor of adverse outcomes in HF, including increased hospitalization rates and mortality [9, 10]. However, the precise mechanisms underlying this association are poorly understood.

A potential biomarker that may shed light on the link between HF, sarcopenia, and adverse outcomes is B-type natriuretic peptide (BNP). BNP is a cardiac hormone produced in response to increased pressure and volume overload, which are hallmarks of HF [11]. Elevated levels of BNP are a well-established diagnostic and prognostic marker for HF, as they reflect the severity of underlying cardiac dysfunction [11, 12]. BNP levels tend to be higher in individuals with muscle wasting and sarcopenia, indicating a potential link between the two conditions [13–15]. Similarly, NT-proBNP, a biologically inactive derivative of BNP, is another biomarker that is commonly used to assess HF severity [12, 16]. However, the relationship between NT-proBNP and sarcopenia is less clear.

Furthermore, cardiac cachexia is a syndrome characterized by the loss of muscle mass and fat, which can occur in the presence of chronic HF [17]. Cardiac cachexia-induced weight loss may also contribute to the development of sarcopenia [18]. This loss of lean mass is often accompanied by an increase in circulating levels of BNP and NT-proBNP [17, 19, 20], highlighting the complex relationship between HF and skeletal muscle wasting, and the need to elucidate the mechanisms behind them.

Given the potential impact of sarcopenia on HF outcomes, it is important to explore potential links that could further explain the relationship between sarcopenia and HF. This systematic review and meta-analysis aims to compare the differences in BNP and NT-proBNP levels in patients with HF, with vs. without sarcopenia, and low vs. higher values of appendicular skeletal muscle mass (ASM), investigating sarcopenia as a potential contributor to aggravated HF states.

## Methods

The revised 2020 Preferred Reporting Items for Systematic Reviews and Meta-Analyses (PRISMA) criteria were followed to conduct this systematic review and meta-analysis [21]. The protocol has been registered in the International Prospective Register of Systematic Reviews (PROSPERO) (CRD42023418465).

### Search strategy

From the beginning until May 2023, PubMed, Scopus, Web of Science, and Cochrane Library were searched independently by K.P and J.M. The search phrases “(heart failure OR ejection fraction) AND (sarcopeni* OR appendicular skeletal muscle OR appendicular lean mass OR skeletal muscle mass OR “ASMI” OR muscle mass)” were employed. Before submission, searches were conducted once more to find any other studies that matched our inclusion criteria.

### Inclusion and exclusion criteria

The following criteria were used to determine which studies should be included: (i) baseline data from observational studies (i.e., cross-sectional, longitudinal, and case-control); (ii) patients with HF regardless of ejection fraction type; (iii) adults with a mean age 50 years old and above; and (iv) clear diagnostic criteria for sarcopenia (i.e., EWGSOP, AWGS, FNIH, or CHS). Published articles were excluded if they (i) were reviews, letters, in vivo or in vitro experiments, commentaries, or posters; (ii) were not published as a full text and in English; and (iii) included participants with mean age <50 years.

### Data extraction and risk of bias

Data on the first author, publication date, country of origin, participants’ age (both with and without sarcopenia), study design, population studied, ejection fraction rate, number of participants, BNP and NT-proBNP levels, definition of sarcopenia, levels of ASM, body composition assessment tool, and reported comorbidities were all extracted independently by two authors (K.P and J.M). Two authors assessed the methodological quality of the studies using three separate tools for cross-sectional population-based studies and cohort-based studies. These checklists all appraise the validity, results, and generalizability of the studies. The tools thoroughly examined the impact of confounders in the quality of results and conclusions. The binary AXIS checklist was used to assess the quality of cross-sectional studies, consisting of 20 questions divided into (1) Introduction, (2) Methods, (3) Results, (4) Discussion, and (5) Other [22]. The risk of bias in cohort studies was assessed utilizing the Cochrane Tool to Assess Risk of Bias in Cohort Studies. Risk of bias appraisal included the assessment of bias domains such as: (1) cohort selection, (2) assessment of exposure, (3) outcome of interest absent at the start of the study, (4) adjustment of prognostic factors, (5) assessment of prognostic factors, (6) assessment of outcome, (7) adequate follow up, and (8) co-intervention similarities between groups. According to the scoring system, study quality was defined as low risk of bias, some concerns, or high risk of bias.

### Statistical analysis

To determine mean differences (MDs) regarding the levels of BNP and NT-proBNP, quantitative data were handled as continuous measurements, and changes in outcomes from patients with sarcopenia and no sarcopenia were compared between groups. The method "standard deviation (SD) = width of IQR/1.35" was used to roughly calculate the missing SDs when studies reported the interquartile range (IQR). In case 95% confidence intervals (95% CIs) were available, SDs were obtained using the equation “SD= √N x (Upper limit of CI – Lower limit of CI)/3.92” [23]. The inverse-variance approach and the random-effects model were used to determine statistical significance.

The overlap of their 95% CIs and measures of Cochran's Q (Chi-square test) and I^2^ were used to analyze the statistical heterogeneity of outcome data across various studies. Low heterogeneity was defined as I^2^ of 30% to 49%, moderate heterogeneity as I^2^ of 50% to 74%, and high heterogeneity as I^2^ of 75% and above [24]. Age, sarcopenia definition, and geographic location were considered in subgroup analyses. Additionally, sensitivity analyses that discounted the impact of comorbidities and/or the significant differences in left ventricular ejection fraction (LVEF) rates between sarcopenia and no sarcopenia groups on outcome measurements, in accordance with the risk of bias of the included cohort studies, were carried out to assess the robustness of the reported statistical results. In relation to ASM, sensitivity analysis was also carried out regarding its definition (low vs. high/normal). The meta-analyses were performed utilizing the programme Review Manager (RevMan 5.4.1). Statistical significance was defined as a *p* value <0.05.

## Results

### Search results

The initial literature search provided 1626 publications. Following the exclusion of duplicates and abstracts, 25 full texts were identified as eligible for inclusion in the systematic review and meta-analysis. Of these 25 studies, six studies were dismissed due to similar cohorts [25–30] with data on participants included in more recent studies that were inserted in our research, one study had participants with a mean age below 50 years old [31], one study had insufficient data on sarcopenia and our outcomes of interest [32], and one study included patients with non-severe or no sarcopenia [33].

**Fig. 1:**
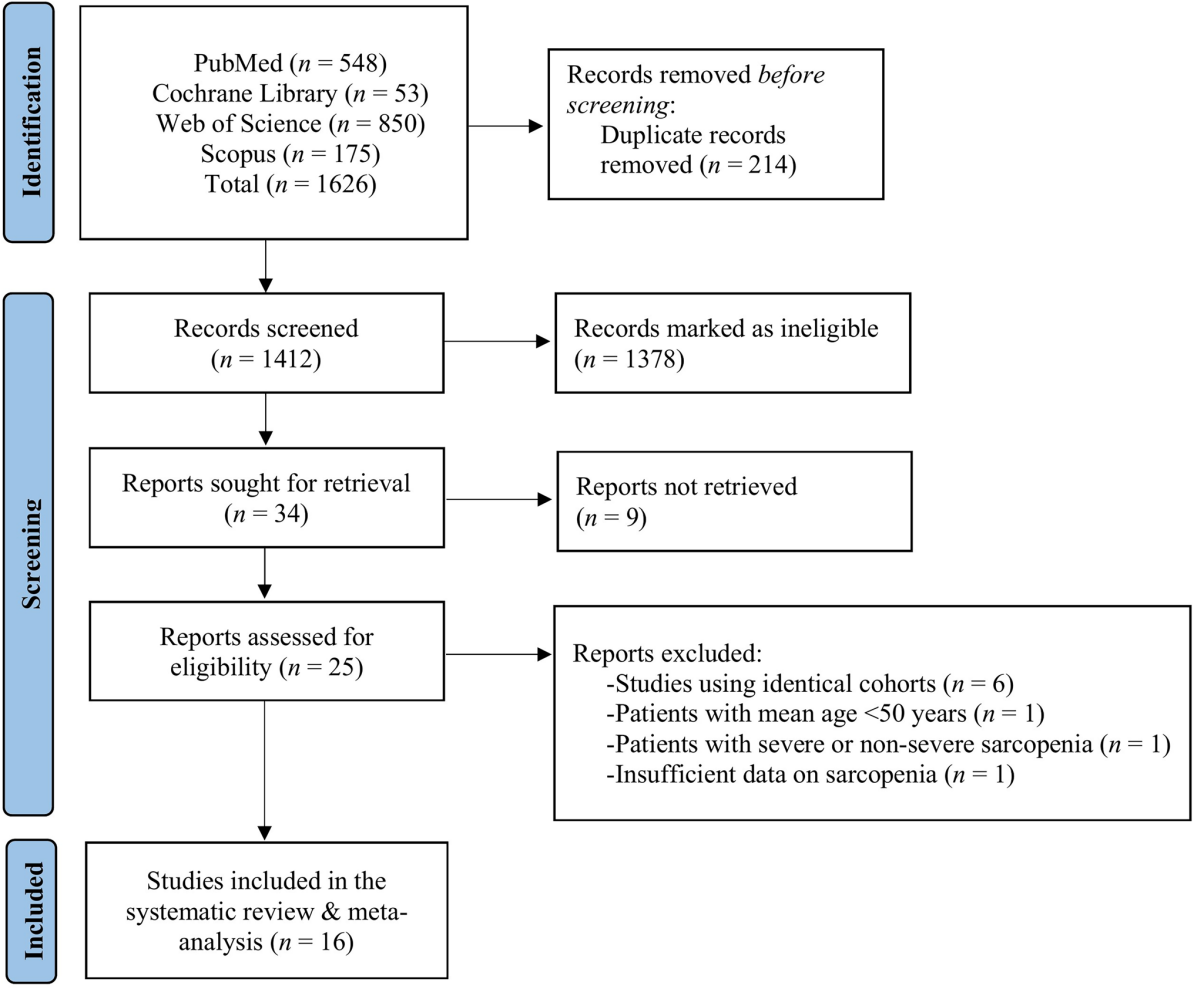
Literature search of the included studies

In total, 16 studies [10, 13, 20, 34–46] were included in this systematic review and meta-analysis exploring the relationship of BNP and NT-proBNP with sarcopenia vs. without sarcopenia and low ASM vs. higher ASM in HF (Fig. 1). Particularly, as exposures, nine studies explored this relationship using sarcopenia [34–38, 40, 41, 45, 46], and seven studies using low ASM [10, 13, 20, 39, 42–44]. Characteristics of the included studies are summarised in Tables S1 and S2.

### Data transformation

Calculation of missing mean (SD) values was conducted in three studies related to sarcopenia and NT-proBNP [35, 37, 46], four studies related to sarcopenia and BNP [34, 37, 38, 40], one study related to ASM and NT-proBNP [10], and five studies in relation to ASM and BNP [13, 39, 42–44].

### BNP levels in sarcopenia vs. no sarcopenia in HF

**Fig. 2:**
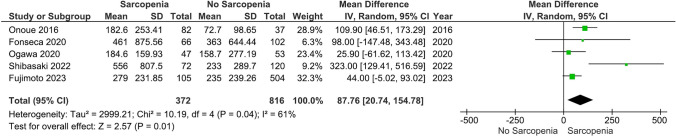
Effects of sarcopenia vs. no sarcopenia on BNP levels in HF

Our main analysis (*k* = 5; 372 subjects with sarcopenia and 816 subjects without sarcopenia) showed that sarcopenia was associated with significantly higher levels of BNP (MD: 87.76, 95% CI 20.74–154.78, *I*^2^ = 61%, *P* = 0.01) (Fig. 2).

Sensitivity analysis based on the exclusion of one study [45] for which patients with sarcopenia had a greater prevalence of hemodialysis did not alter the findings (MD: 63.03, 95% CI 23.51–102.54, *I*^2^ = 13%, *P* = 0.002) (Fig. S1). Identical results were displayed after the exclusion of one study [40] due to a higher risk of bias (MD: 89.16, 95% CI 15.29–163.03, *I*^2^ = 70%, *P* = 0.02) (Fig. S2). Conversely, excluding a study using the Ishii index to define sarcopenia [34], our analysis showed insignificant changes between groups (MD: 87.30, 95% CI − 6.41 – 181.01, *I*^2^ = 63%, *P* = 0.07) (Fig. S3).

### NT-proBNP levels in sarcopenia vs. no sarcopenia in HF

**Fig. 3:**
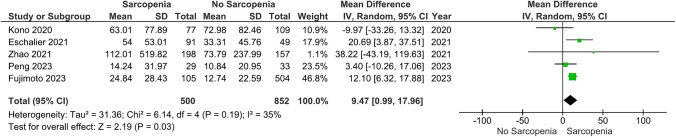
Effects of sarcopenia vs. no sarcopenia on NT-proBNP levels in HF

Our main analysis (*k* = 5; 500 subjects with sarcopenia and 852 subjects without sarcopenia) showed that sarcopenia was associated with significantly higher levels of NT-proBNP (MD: 947.45, 95% CI 98.97 – 1795.93, *I*^2^ = 35%, *P* = 0.03) (Fig. 3).

Sensitivity analysis based on the exclusion of two studies [35, 36] for which patients with sarcopenia had a greater prevalence of stroke and coronary heart disease (MD: 590.23, 95% CI − 481.05–1661.50, *I*^2^ = 53%, *P* = 0.28) (Fig. S4). When we excluded the study with the highest risk of bias [41], people with sarcopenia exhibited statistically higher levels of NT-proBNP vs. those without sarcopenia (MD: 1178.32, 95% CI 671.53–1685.10, *I*^2^ = 0%, *P* < 0.01) (Fig. S5). Similarly, given that in the study by Kono *et al.* (2020) [41] those with sarcopenia had significantly higher ejection fraction rates compared to participants with no sarcopenia, a sensitivity analysis was also performed (MD: 1178.32, 95% CI 671.53–1685.10, *I*^2^ = 0%, *P* < 0.01) (Fig. S6).

### BNP levels in participants with low vs. higher levels of ASM

**Fig. 4:**
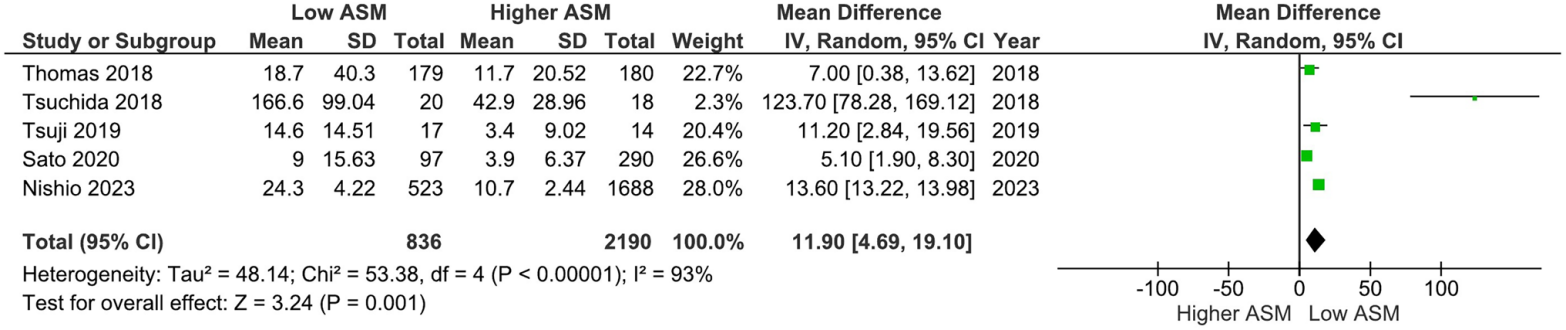
Effects of low ASM on BNP levels in HF

Our main analysis (*k* = 5; 836 subjects with low ASM and 2190 subjects with higher ASM) showed that low ASM was associated with significantly higher levels of BNP, although an increased degree of heterogeneity was observed (MD: 118.95, 95% CI 46.91–191.00, *I*^2^ = 93%, *P* < 0.01) (Fig. 4).

Considering that two studies [43, 44] had very different cut-off values for low ASM, a sensitivity analysis was performed without altering our results (MD: 152.95, 95% CI 44.18–261.72, *I*^2^ = 96%, *P* < 0.01) (Fig. S7). In addition, sensitivity analysis based on a higher prevalence of CKD [39] in subjects with low ASM also showed similar findings (MD: 143.09, 95% CI 23.77–262.41, *I*^2^ = 89%, *P* = 0.02) (Fig. S8). Likewise, in relation to ASM, the exclusion of two studies that had an overall moderate risk of bias [13, 43] did not alter the findings derived from the main analysis regarding BNP levels (MD: 99.38, 95% CI 32.50–166.26, *I*^2^ = 93%, *P* < 0.01) (Fig. S9).

### NT-proBNP levels in participants with low vs. higher levels of ASM

**Fig. 5:**
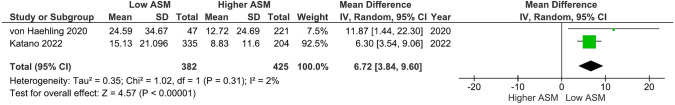
Effects of low ASM on NT-proBNP levels in HF

Our main analysis (*k* = 2; 382 subjects with low ASM and 425 subjects with higher ASM) showed that low ASM was associated with significantly higher levels of NT-proBNP (MD: 672.01, 95% CI 383.72–960.30, *I*^2^ = 2%, *P* < 0.01) (Fig. 5).

### Notes on figures: modifications of forest plot scales

Considering that forest plot scales in RevMan 5.4.1 go up to 1000, we had to modify some of our plots. For example, the mean and SD values of Fig. 4 and Figs. S7-S9 were divided by 10, while Fig. 3, 5, and Fig.s S4–S6 were divided by 100.

### Risk of bias of included studies

The overall quality of the included studies exploring the impact of sarcopenia was considered moderate, albeit most studies had a low risk (Table S3, Table S4). In addition, the quality of the included studies exploring the impact of low ASM was considered low (Table S5).

## Discussion

In this systematic review and meta-analysis of 16 studies, we found that patients with HF and sarcopenia, and those with low ASM have higher plasma levels of BNP and NT-proBNP compared to patients without sarcopenia and higher ASM, respectively.

Although the physiological rationale underpinning the reason sarcopenia and low ASM could lead to reduced levels of cardiac function markers is limited, research has indicated that this effect may be mediated by alterations of sex steroid hormones. In particular, higher androgen concentrations have been linked to increased ASM and lower natriuretic peptide release [47–49], whereas oestrogens, which are associated with decreased ASM, may raise natriuretic peptide levels [50]. However, to date, evidence behind the biological processes justifying these findings is lacking, and research around this area is warranted. Furthermore, skeletal muscle growth mediators (i.e., Akt1, Follistatin-1) with potentially cardioprotective impact via regulation of endothelial cell function and blood vessel growth in skeletal muscle could partially describe this phenomenon [51–53]. Mechanistically, follistatin may downregulate transforming growth factor beta (TGF-β) member activity and the phosphorylation of Smad3 [54, 55]; critical promoters of muscle atrophy [56]. Nevertheless, human research has shown that increased follistatin production is linked to left ventricular adverse remodelling rather than improved left ventricular function [57]. Understanding the relationship between muscular dysfunction and heart failure may rely on fully clarifying the link between sarcopenia and elevated natriuretic peptide levels.

Moreover, individuals exhibiting substantial weight loss display elevated NT-proBNP levels compared to people with stable weight [58]. Interestingly, patients with HF and cachexia have elevated levels of BNP compared to bodyweight-stable patients [19], which have been linked to altered epicardial adipose tissue metabolism and greater regional fat thickness [59]. HF may induce the production of adiponectin and promote lipolysis through elevated levels of natriuretic peptides [60], and concomitantly, may also increase plasma levels of myostatin and proinflammatory cytokines, which are linked to muscle wasting [14, 61]. The above observations speculate that some patients with HF may have a dysregulated inflammatory, myostatin, lipolytic, and even appetite profile compared to other patients, which could in part explain our findings.

### Strengths and limitations

This is the first study attempting to systematically examine the association of sarcopenia with BNP and NT-proBNP in patients with HF. Our meta-analysis employed multiple subgroup and sensitivity analyses, including controlling for comorbidities and LVEF, utilizing both sarcopenia and ASM to provide greater consistency.

Our study, however, has several limitations. We could not perform additional analyses to explore the impact of sex and whether the type of HF [with reduced (HFrEF) or preserved (HFpEF) ejection fraction] would exhibit different outcomes. Additionally, although secondary sarcopenia in patients with HF may lead to increased levels of natriuretic peptides, it is worth considering the additional impact of age-related loss of muscle mass and strength. In the included studies, sarcopenic groups were notably older than patients without sarcopenia, implying that age may be a significant contributor to our results. Albeit, ageing is linked to a higher prevalence of diastolic dysfunction, the participants included in the analysis had comparable levels of LVEF following adjustment via sensitivity analysis, highlighting that ageing could influence BNP and NT-proBNP in other ways. In addition, although both natriuretic peptides are closely related, their distribution among studies was unequal. Considering this, the non-normal distribution of biomarkers may influence the robustness of our analyses. Moreover, natriuretic peptides are some of several biomarkers of cardiac function, therefore, investigation of more biomarkers may be warranted. It is noteworthy that the studies included did not include details of the natriuretic peptide assays used, although it seems reasonable to assume these were all clinical diagnostic assays. There are several manufacturers who supply NT-proBNP and BNP assays, each with different performance characteristics. While the effect of different assay methodologies is likely to be negligible, we were not able to assess this. All results were, however, reported in standard units (ng/L or pg/ml). Added to this, it is imperative to highlight that our analytical approach deviated from utilizing raw mean (SD) values. Instead, we opted for the transformation of median (IQR) values in our analyses. Furthermore, medications could influence circulating levels of natriuretic peptides [62], however, two studies did not report details of medications [36, 45]. Finally, considering the limited observational studies, we could not extrapolate data pertinent to other individual measures of sarcopenia such as handgrip strength, gait speed, or physical performance.

## Conclusions

Sarcopenia and low ASM are associated with higher plasma levels of BNP and NT-proBNP in patients with HF. In clinical practice, assessment of sarcopenia in patients with HF could potentially be complementary to such biomarkers and guide therapeutic interventions. Future research is required to investigate whether sarcopenia could lead to dysregulated biomarkers of cardiac function.

## Supplementary Information

Below are links to the electronic supplementary material.

Supplementary file1 (JPG 271 KB) Figure S1. Effects of sarcopenia vs. no sarcopenia on BNP levels in HF

Supplementary file2 (JPG 267 KB) Figure S2. Effects of sarcopenia vs. no sarcopenia on BNP levels in HF

Supplementary file3 (JPG 265 KB) Figure S3. Effects of sarcopenia vs. no sarcopenia on BNP levels in HF

Supplementary file4 (JPG 258 KB) Figure S4. Effects of sarcopenia vs. no sarcopenia on NT-proBNP levels

Supplementary file5 (JPG 259 KB) Figure S5. Effects of sarcopenia vs. no sarcopenia on NT-proBNP levels

Supplementary file6 (JPG 257 KB) Figure S6. Effects of sarcopenia vs. no sarcopenia on NT-proBNP levels

Supplementary file7 (JPG 258 KB) Figure S7. Effects of low ASM on BNP levels in HF excluding two

Supplementary file8 (JPG 256 KB) Figure S8. Effects of low ASM on BNP levels in HF excluding one study

Supplementary file9 (JPG 253 KB) Figure S9. Effects of low ASM on BNP levels in HF based on risk of

Supplementary file10 (DOCX 21 KB)

Supplementary file11 (DOCX 17 KB)

Supplementary file12 (DOCX 15 KB)

Supplementary file13 (DOCX 22 KB)

Supplementary file14 (DOCX 15 KB)

## Data Availability

Data is available upon request.
