# Supplementary figures and images for: Sarcopenia is linked to higher levels of B-type natriuretic peptide and its N-terminal fragment in heart failure: a systematic review and meta-analysis
Source: Eur Geriatr Med. 2024 Mar 8;15(4):893–901. doi: 10.1007/s41999-024-00950-x (PMC11377361; doi:10.1007/s41999-024-00950-x)

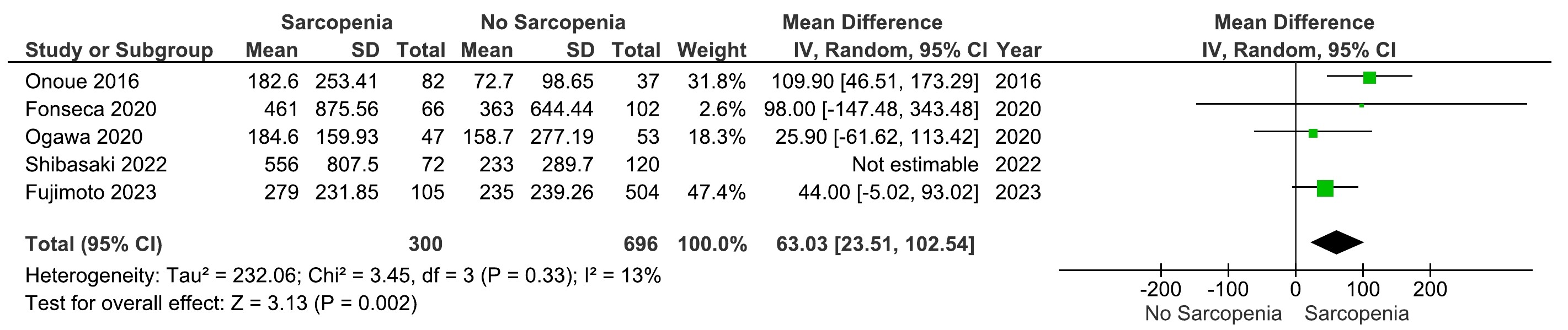

Supplement: Supplementary file 1 — Supplementary file1 (JPG 271 KB) Figure S1. Effects of sarcopenia vs. no sarcopenia on BNP levels in HF [file 41999_2024_950_MOESM1_ESM.jpg]

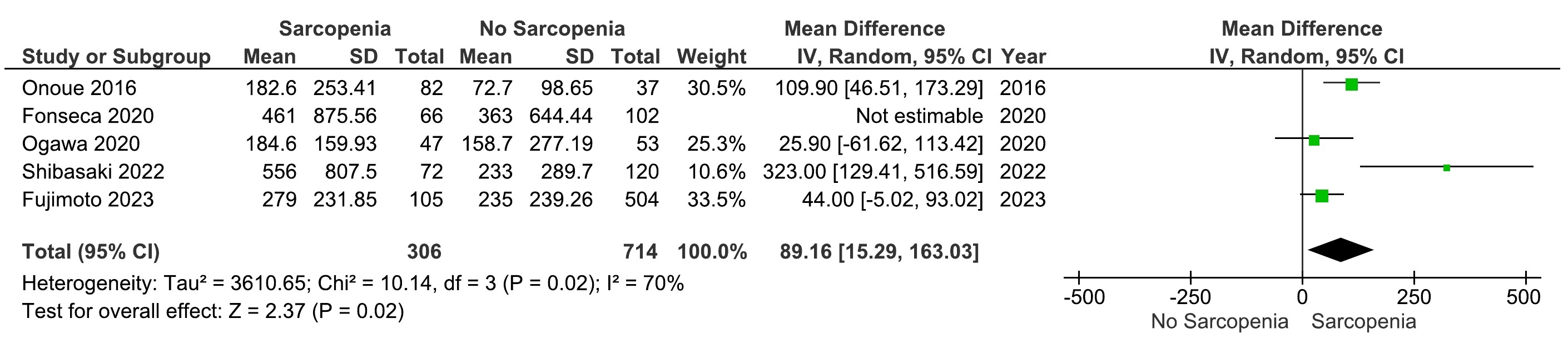

Supplement: Supplementary file 2 — Supplementary file2 (JPG 267 KB) Figure S2. Effects of sarcopenia vs. no sarcopenia on BNP levels in HF [file 41999_2024_950_MOESM2_ESM.jpg]

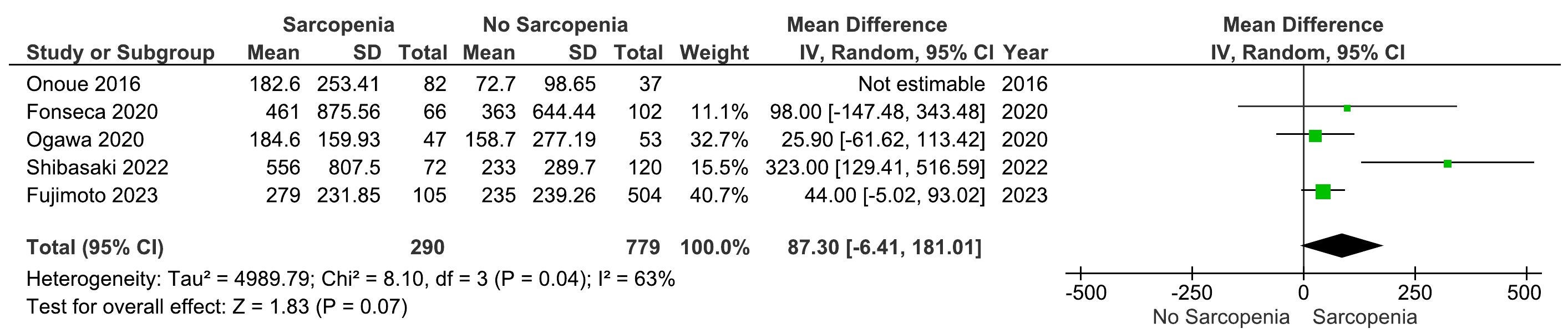

Supplement: Supplementary file 3 — Supplementary file3 (JPG 265 KB) Figure S3. Effects of sarcopenia vs. no sarcopenia on BNP levels in HF [file 41999_2024_950_MOESM3_ESM.jpg]

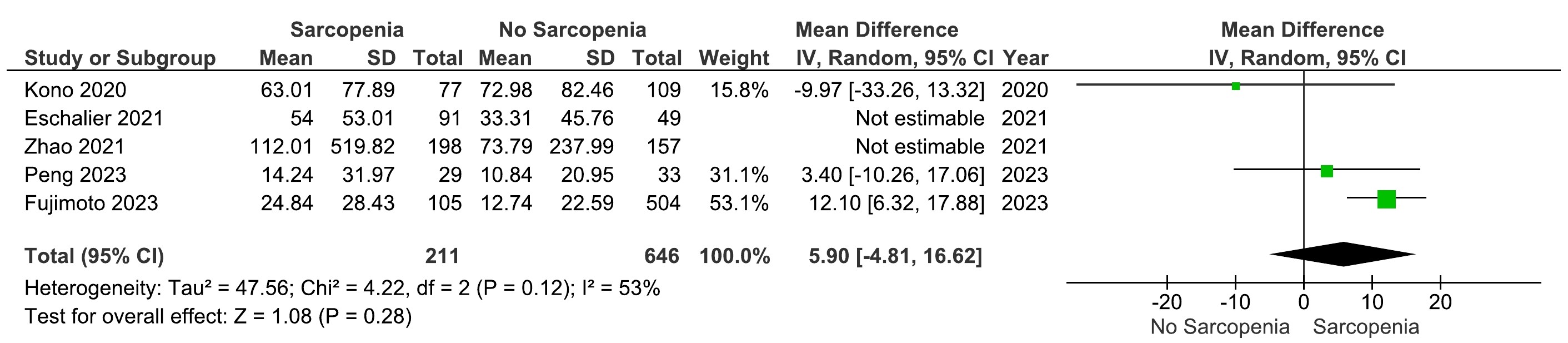

Supplement: Supplementary file 4 — Supplementary file4 (JPG 258 KB) Figure S4. Effects of sarcopenia vs. no sarcopenia on NT-proBNP levels [file 41999_2024_950_MOESM4_ESM.jpg]

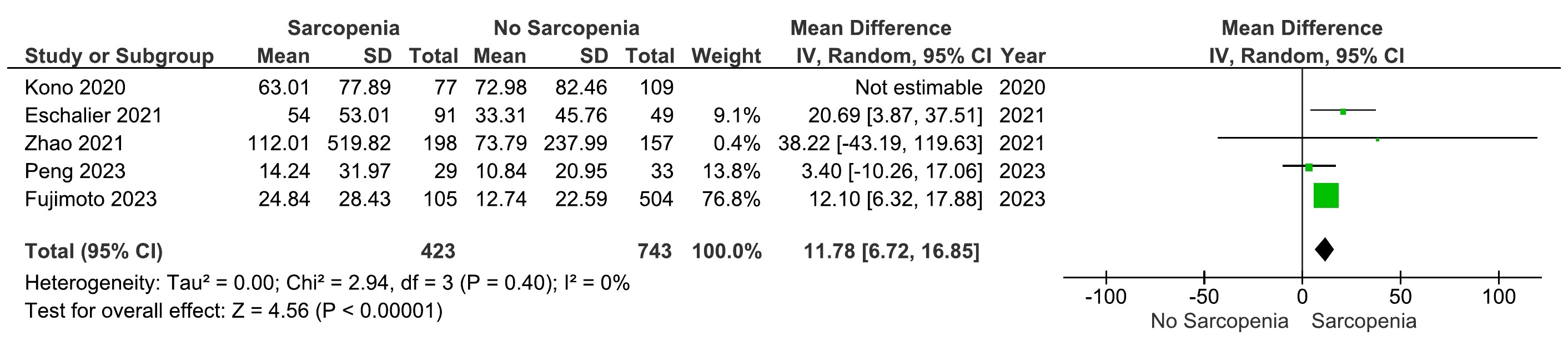

Supplement: Supplementary file 5 — Supplementary file5 (JPG 259 KB) Figure S5. Effects of sarcopenia vs. no sarcopenia on NT-proBNP levels [file 41999_2024_950_MOESM5_ESM.jpg]

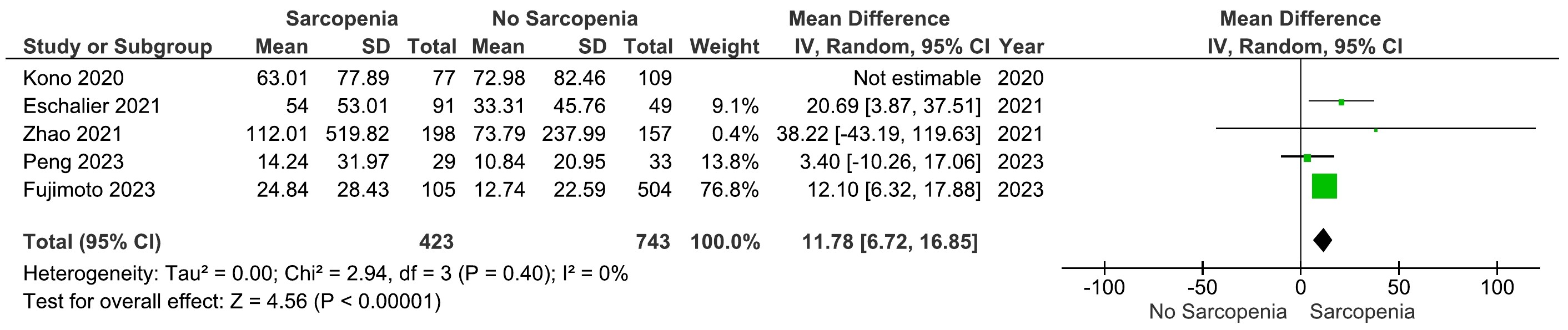

Supplement: Supplementary file 6 — Supplementary file6 (JPG 257 KB) Figure S6. Effects of sarcopenia vs. no sarcopenia on NT-proBNP levels [file 41999_2024_950_MOESM6_ESM.jpg]

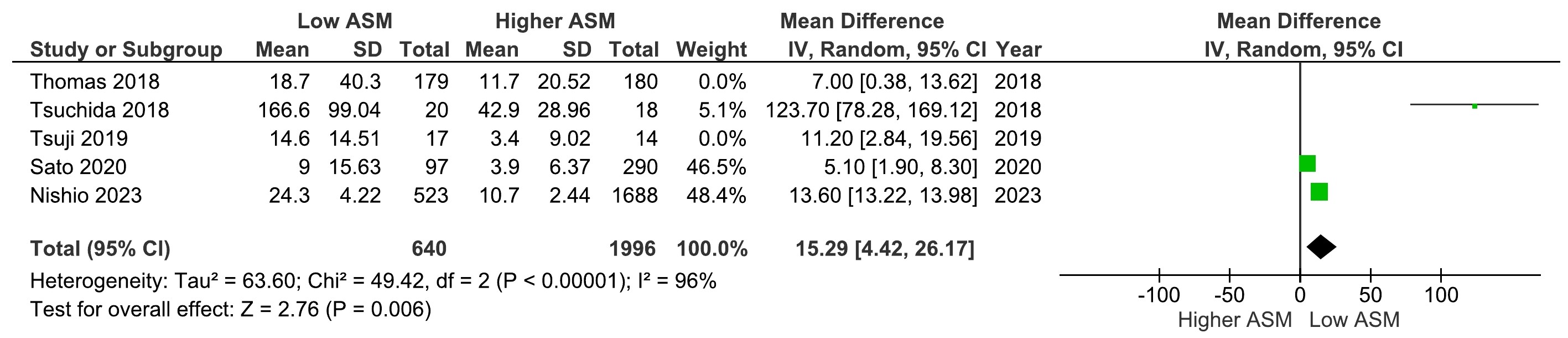

Supplement: Supplementary file 7 — Supplementary file7 (JPG 258 KB) Figure S7. Effects of low ASM on BNP levels in HF excluding two [file 41999_2024_950_MOESM7_ESM.jpg]

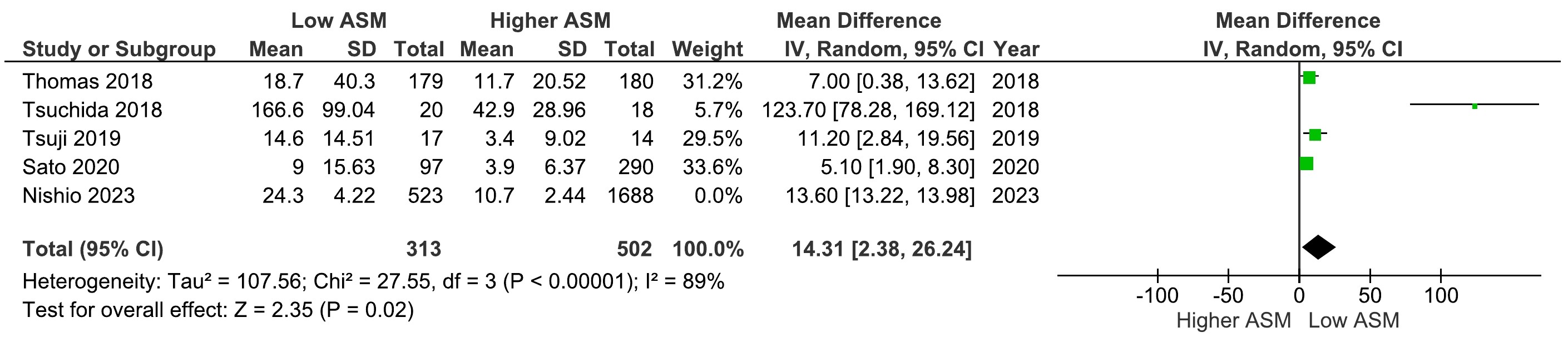

Supplement: Supplementary file 8 — Supplementary file8 (JPG 256 KB) Figure S8. Effects of low ASM on BNP levels in HF excluding one study [file 41999_2024_950_MOESM8_ESM.jpg]

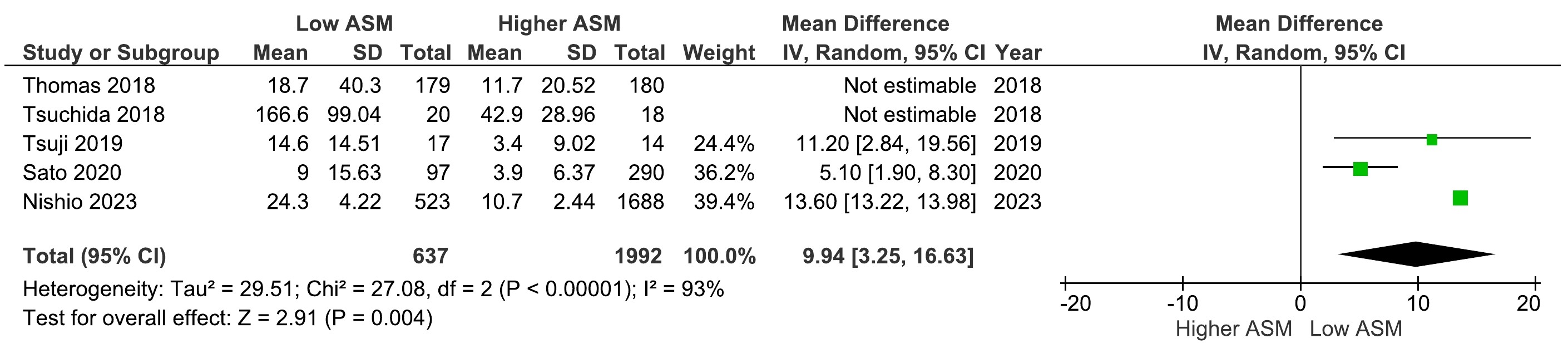

Supplement: Supplementary file 9 — Supplementary file9 (JPG 253 KB) Figure S9. Effects of low ASM on BNP levels in HF based on risk of [file 41999_2024_950_MOESM9_ESM.jpg]
