# Supplementary material for: Sarcopenia is linked to higher levels of B-type natriuretic peptide and its N-terminal fragment in heart failure: a systematic review and meta-analysis
Source: Eur Geriatr Med. 2024 Mar 8;15(4):893–901. doi: 10.1007/s41999-024-00950-x (PMC11377361; doi:10.1007/s41999-024-00950-x)
Supplement: Supplementary file 10 — Supplementary file10 (DOCX 21 KB) [file 41999_2024_950_MOESM10_ESM.docx]

**Table S1.** Study and participant characteristics of the included studies using sarcopenia in the systematic review and meta-analysis.

| **Study**  **Year**  **Country** | **Sarcopenia or muscle dysfunction definition** | **Total *n*  (M/F)** | **HF with sarcopenia or muscle dysfunction** | | | **HF without sarcopenia or muscle dysfunction** | | | **Reported differences in comorbidities** | **Sarcopenia**  **components**  **used** | **Body Composition Assessment Tool** |
| --- | --- | --- | --- | --- | --- | --- | --- | --- | --- | --- | --- |
|  |  |  | ***n* (M/F)** | **Age**  **(SD)** | **LVEF**  **(%)** | **n**  **(M/F)** | **Age**  **(SD)** | **LVEF**  **(%)** |  |  |  |
| Fujimoto  2023  Japan  (NT-proBNP) | AWGS 2019 | 609  (357/252) | 105 (78/27) | 82 (75, 87) | 40 (29, 58) | 504 (279/225) | 79 (73, 85) | 46 (33, 60) | AF, CAD, COPD, T2D, Hypertension, Malignancy | ASM (≤7.0 kg/m2 males; ≤5.7 kg/m2 females); HGS (<28 kg males; <18 kg females); GS (≤1 m/s) or SPPB (≤9) or 5TCS ≥12 seconds) | BIA |
| Fujimoto 2023  Japan  (BNP) | AWGS 2014 | 609  (357/252) | 105 (78/27) | 82 (75, 87) | 40 (29, 58) | 504 (279/225) | 79 (73, 85) | 46 (33, 60) | AF, CAD, COPD, T2D, Hypertension, Malignancy | ASM (≤7.0 kg/m2 males; ≤5.7 kg/m2 females); HGS (<26 kg males; <18 kg females); GS (≤0.8 m/s) | BIA |
| Peng  2023  China | AWGS 2019 | 62  (37/25) | 29 (13/16) | 75.14 ± 8.18 | 55.00 (38.00, 60.00) | 33 (24/9) | 71.76 ± 7.93 | 57.00 (39.50, 61.50) | - | ASM (<7.0 kg/m2 males; <5.7 kg/m2 females); HGS (<28 kg males; <16 kg females); GS (<1 m/s) | BIA |
| Shibasaki 2022  Japan | AWGS 2014 | 192  (126/66) | 72 (43/29) | 73.8 ± 8.8 | 56.2 ± 13.3 | 120 (83/37) | 67.0 ± 10.1 | 59.4 ± 12.3 | T2D, Hypertension, Dyslipidemia, Hemodialysis | ASM (<7.0 kg/m2 males; <5.7 kg/m2 females); HGS (<26 kg males; <18 kg females); GS (<0.8 m/s) | BIA |
| Eschalier 2021  France | EWGSOP1 | 140  (82/58) | 91 (54/37) | 78.2 ± 9.0 | 42.8 ± 14.7 | 49 (28/21) | 71.4 ± 10.9 | 40.7 ± 14.0 | AF, T2D, Hypertension, Dyslipidemia, COPD, Vascular arteriopathy, Pulmonary Infection | ASM (<10.75 kg/m2 males; <6.75 kg/m2 females); HGS (<30 kg males; <20 kg females); <0.8 m/s) | BIA |
| Zhao  2021  China | AWGS 2014 | 355  (207/148) | 198 (105/93) | 73.58 (10.21) | - | 157 (102/55) | 67.76 (7.08) | - | Hypertension, Stroke, CAD, T2D | ASM (<7.0 kg/m2 males; <5.7 kg/m2 females); HGS (<26 kg males; <18 kg females); GS (<0.8 m/s) | BIA |
| Fonseca 2020  Brazil | EWGSOP1 | 355  (207/148) | 66 (66/0) | 60 (55, 63) | 25 (21, 34) | 102 (102/0) | 56 (51, 62) | 27 (22, 33) | - | ASM (Lowest 20th percentile) based on BMI; HGS (Lowest 20th percentile) based on BMI | DXA |
| Kono  2020  Japan | Japanese Geriatrics Society | 186  (81/105) | 77 (15/62) | 85.6 (6.9) | 62.0 (16.1) | 109 (66/43) | 75.3 (9.04) | 45.8 (17.7) | T2D, Hypertension, Stroke, COPD | BMI (<18 kg/m2); HGS (<26 kg males; <18 kg females); GS (<0.8 m/s) | - |
| Ogawa 2020  Japan | AWGS 2014 | 100  (62/38) | 47 (25/22) | 80.0 (75.0, 84.0) | 48.0 (30.0, 66.0) | 53 (37/16) | 75.0 (68.0, 79.5) | 40.5 (30.0, 60.8) | Stroke, AF, T2D, Hypertension, Dyslipidemia | ASM (<7.0 kg/m2 males; <5.7 kg/m2 females); HGS (<26 kg males; <18 kg females); GS (<0.8 m/s) | BIA |
| Onue  2016  Japan | Ishii index | 119  (73/46) | 82 (53/29) | 77.6 ± 5.4 | 53.8 ± 12.3 | 37 (20/17) | 72.0 ± 5.9 | 58.8 ± 11.8 | T2D, Hypertension, Dyslipidemia | Males: 0.62 × (age − 64) − 3.09 × (grip strength − 50) − 4.64 × (calf circumference − 42); Females: 0.80 × (age − 64) − 5.09 × (grip strength − 34) − 3.28 × (calf circumference − 42); Sarcopenia score ≥ 105 in men and ≥120 in women | - |

Abbreviations: 5TCS, 5-time chair stand; AF, atrial fibrillation; ASM, appendicular skeletal muscle; AWGS, Asian Working Group for Sarcopenia; BIA, bioelectrical impedance; CAD, coronary artery disease; COPD, chronic obstructive pulmonary disease; DXA, dual x-ray absorptiometry; EWGSOP, European Working Group for Sarcopenia in Older People; F, females; GS, gait speed; HGS, handgrip strength; M, males; SD, standard deviation; T2D, type 2 diabetes.

Data are expressed as mean ± SD.

Data are expressed as median (IQR).
