# Supplementary material for: Sarcopenia is linked to higher levels of B-type natriuretic peptide and its N-terminal fragment in heart failure: a systematic review and meta-analysis
Source: Eur Geriatr Med. 2024 Mar 8;15(4):893–901. doi: 10.1007/s41999-024-00950-x (PMC11377361; doi:10.1007/s41999-024-00950-x)
Supplement: Supplementary file 11 — Supplementary file11 (DOCX 17 KB) [file 41999_2024_950_MOESM11_ESM.docx]

**Table S2.** Study and participant characteristics of the included studies using ASM in the systematic review and meta-analysis.

| **Study**  **Year**  **Country** | **Total *n*  (M/F)** | **HF with sarcopenia or muscle dysfunction** | | | | | **HF without sarcopenia or muscle dysfunction** | | | | | **Reported differences in comorbidities** | **Sarcopenia**  **components**  **used** | **Body Composition Assessment Tool** |
| --- | --- | --- | --- | --- | --- | --- | --- | --- | --- | --- | --- | --- | --- | --- |
|  |  | ***n* (M/F)** | **Age**  **(SD)** | **LVEF**  **(%)** | **BNP** | **NT-proBNP** | **n**  **(M/F)** | **Age**  **(SD)** | **LVEF**  **(%)** | **BNP** | **NT-proBNP** |  |  |  |
| Katano 2022 Japan | 539 (307/232) | 335 (201/134) | 73 (66, 81) | 45.9 (32.4, 62.0) | - | 1513 (666, 3514) | 204 (106/98) | 72 (60, 82) | 51.7 (36.2, 64.0) | - | 883 (365, 1931) | Hypertension, Dyslipidemia, T2D, AF, Cancer | ASMI (<7.00 kg/m2 for men  and <5.40 kg/m2 for women) | DXA |
| von Haehling (SICA-HF) 2020 Germany | 268 (211/57) | 47 (45/2) | 71.94 ± 8.60 | All: 39.00 ± 13.25 | - | 2459 ± 3467 | 221 (166/55) | 66.12 ± 11.04 | All: 39.00 ± 13.25 | - | 1272 ± 2469 | - | ASMI (< 7.26 kg/m2 for men and < 5.45 kg/m2 for women) | DXA |
| Nishio 2023 Japan | 2211 (1802/409) | 523 (436/87) | 74.0 ± 8.8 | 58.4 ± 13.7 | 243 (215–272); SD: 42.2 | - | 1688 (1366/322) | 65.5 ± 10.9 | 61.3 ± 11.6 | 107 (91–124); SD: 24.4 | - | T2D, Hypertension, CKD, Dyslipidemia | ASMI (< 7.30 kg/m2 for men and < 5.0 kg/m2 for women) | - |
| Sato 2020 Japan | 387 (315/69) | 97 (79/15) | 74 (9) | 43 (12) | 90 (43, 254); SD: 156.3 | - | 290 (236/54) | 63 (13) | 47 (11) | 39 (17, 103); SD: 63.7 | - | Hypertension, T2D, Dyslipidemia | ASMI (≤ 6.64 kg/m2 for men and ≤ 5.06 kg/m2 for women) | DXA |
| Tsuji 2019 Japan | 31 (20/11) | 17 (10/7) | 72.6 ± 8.6 | All: 47.6 ± 14 | 146 (74.1–270); SD: 145.1 | - | 14 (10/4) | 71.1 ± 8.5 | All: 47.6 ± 14 | 34.0 (17.6–139.4); SD: 90.2 | - | - | Low SMI: 8.7 ± 1.5 kg/m2; High SMI: 9.4 ± 1.4 kg/m2 | BIA |
| Thomas 2018 USA | 359 (260/99) | 179 (95/84) | 58.0 (15.2) | 38 (16) | 187 (40-584); SD: 403 | - | 180 (165/15) | 53.8 (12.2) | 34 (16) | 117 (30-307); SD: 205.2 | - | T2D | Low LBMI (≤ 19.1 kg/m2); High LBMI (> 19.1 kg/m2) | DXA |
| Tsuchida 2018 Japan | 38 (25/13) | 20 (16/4) | 77.9 ± 9.1 | 45.6 ± 13.8 | 1666 [661, 1998]; SD: 990.4 | - | 18 (9/9) | 72.0 ± 13.1 | 49.6 ± 16.9 | 429.0 [260, 651]; SD: 289.6 | - | T2D, Hypertension, COPD, AF | ASMI (< 6.87 kg/m2 in men and < 5.46 kg/m2 in women) | DXA |

Abbreviations: AF, atrial fibrillation; ASM, appendicular skeletal muscle; BIA, bioelectrical impedance; CKD, chronic kidney disease; COPD, chronic obstructive pulmonary disease; DXA, dual x-ray absorptiometry; F, females; LBMI, lean body mass index; M, males; SD, standard deviation; T2D, type 2 diabetes.

Data are expressed as mean ± SD.

Data are expressed as median (IQR).
