# Supplementary material for: Sarcopenia is linked to higher levels of B-type natriuretic peptide and its N-terminal fragment in heart failure: a systematic review and meta-analysis
Source: Eur Geriatr Med. 2024 Mar 8;15(4):893–901. doi: 10.1007/s41999-024-00950-x (PMC11377361; doi:10.1007/s41999-024-00950-x)
Supplement: Supplementary file 12 — Supplementary file12 (DOCX 15 KB) [file 41999_2024_950_MOESM12_ESM.docx]

**Table S3.** Quality assessment of the nine included cohort-based observational studies exploring the impact of sarcopenia using the Cochrane RoB tool.

| Study | D1 | D2 | D3 | D4 | D5 | D6 | D7 | D8 |
| --- | --- | --- | --- | --- | --- | --- | --- | --- |
| Fujimoto  2023 | Low risk | Low risk | Low risk | Some concerns | Some concerns | Low risk | Some concerns | Low risk |
| Shibasaki 2022 | Low risk | Low risk | Low risk | Some concerns | Low risk | Low risk | Low risk | Low risk |
| Eschalier  2021 | Low risk | Low risk | Low risk | Some concerns | Low risk | Low risk | Low risk | Low risk |
| Fonseca  2020 | Low risk | Low risk | Some concerns | Some concerns | Some concerns | Low risk | Some concerns | Low risk |
| Kono  2020 | Low risk | Low risk | Low risk | Some concerns | Some concerns | Some concerns | Some concerns | Low risk |
| Ogawa  2020 | Low risk | Low risk | Low risk | Some concerns | Low risk | Low risk | Low risk | Low risk |
| Peng  2023 | Low risk | Low risk | Low risk | Some concerns | Low risk | Low risk | Low risk | Low risk |
| Onoue  2016 | Low risk | Low risk | Low risk | Low risk | Low risk | Low risk | Low risk | Low risk |

D1: Selection of exposed vs. non-exposed cohorts drawn from same population; D2: Confidence in assessment of exposure; D3: Confident in outcome of interest not at start of study; D4: Matching exposed and unexposed for all variables associated with outcome of interest; D5: Confident in assessment of presence/absence of prognostic factors; D6: Confident in assessment of outcome; D7: Adequate follow-up of cohorts; D8: Co-intervention similar between groups?
