# Supplementary material for: Sarcopenia is linked to higher levels of B-type natriuretic peptide and its N-terminal fragment in heart failure: a systematic review and meta-analysis
Source: Eur Geriatr Med. 2024 Mar 8;15(4):893–901. doi: 10.1007/s41999-024-00950-x (PMC11377361; doi:10.1007/s41999-024-00950-x)
Supplement: Supplementary file 13 — Supplementary file13 (DOCX 22 KB) [file 41999_2024_950_MOESM13_ESM.docx]

**Table S4**. Quality assessment of the one included cross-sectional study exploring the impact of sarcopenia according to the AXIS tool.

| Study | Q1 | Q2 | Q3 | Q4 | Q5 | Q6 | Q7 | Q8 | Q9 | Q10 | Q11 | Q12 | Q13 | Q14 | Q15 | Q16 | Q17 | Q18 | Q19 | Q20 |
| --- | --- | --- | --- | --- | --- | --- | --- | --- | --- | --- | --- | --- | --- | --- | --- | --- | --- | --- | --- | --- |
| Zhao 2021 | 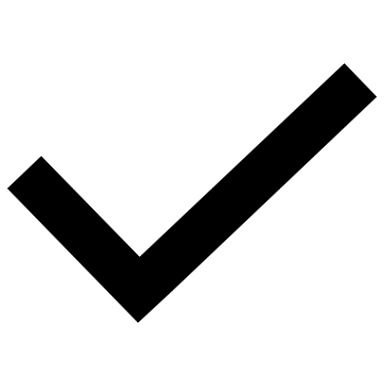 | 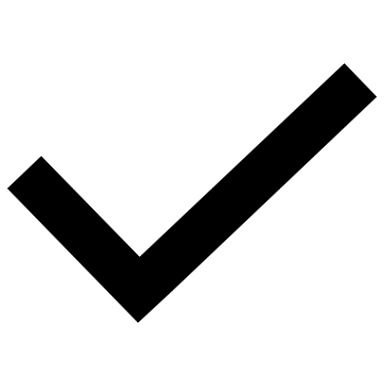 |  | 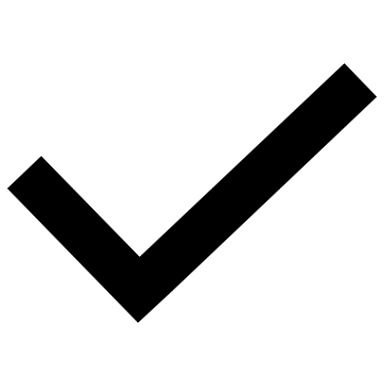 | 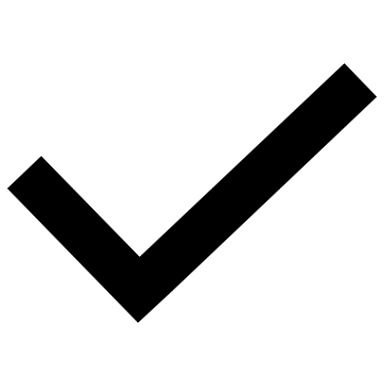 | 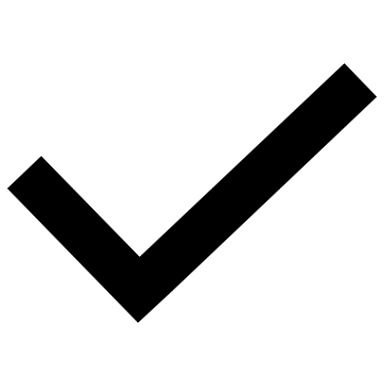 |  | 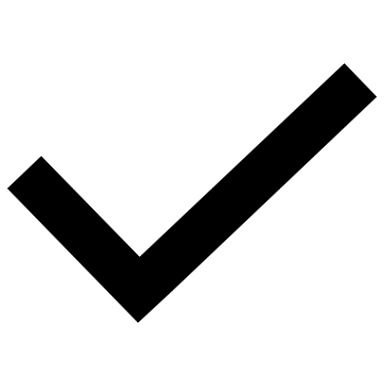 | 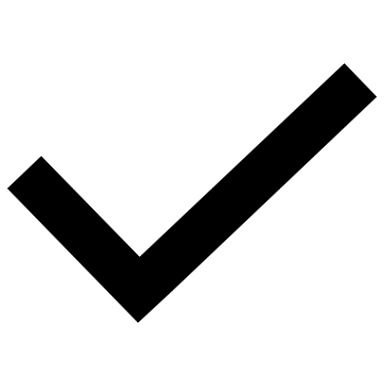 | 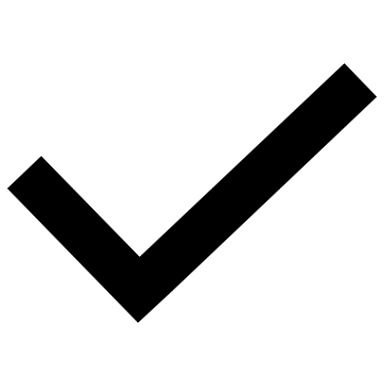 | 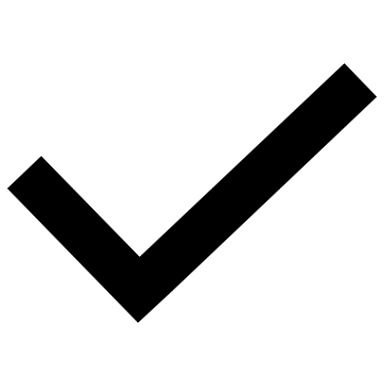 | 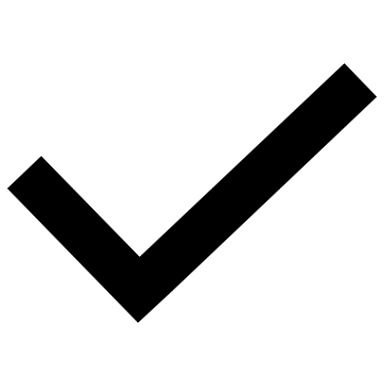 |  |  | 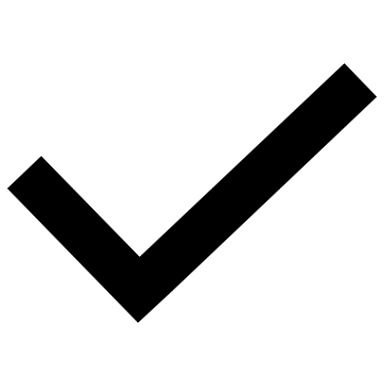 | 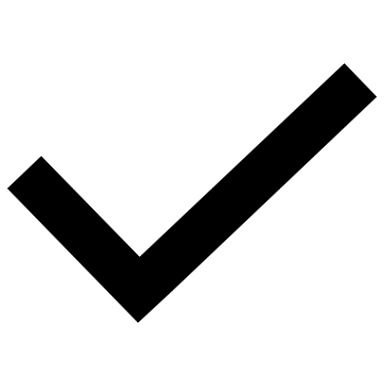 | 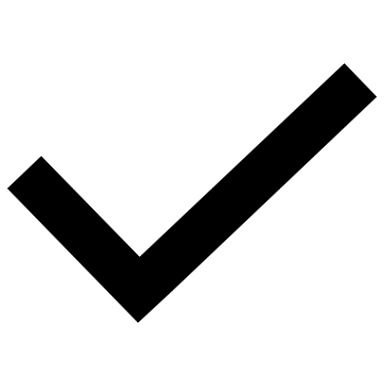 | 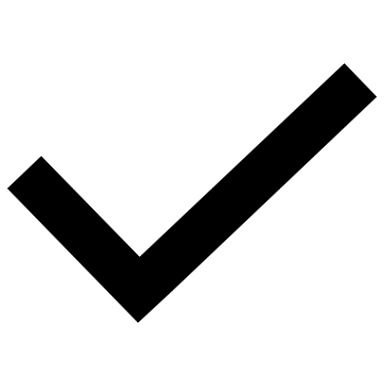 |  | 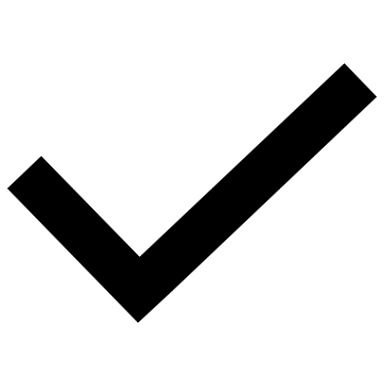 |

Q1: Clear aims; Q2: Appropriate study design; Q3: Justified sample size; Q4: Defined targeted population; Q5: Appropriate population sample; Q6: Representative participant selection; Q7: Non-responders categorization measures; Q8: Measured outcome variables related to aims; Q9: Measured outcome variables with previously trialed measurements; Q10: Clear tools to determine statistical significance; Q11: Described reproducible methods; Q12: Adequately described basic data: Q13; Concerns of response rates for non-responders: Q14; Described information about non-responders: Q15: Internally consistent results; Q16: Presented results for all analyses in methods; Q17: Justified conclusions with results; Q18: Discussed limitations; Q19: Concerns about conflict of interest; Q20: Attained ethical approval or participant consent
