# Supplementary material for: Sarcopenia is linked to higher levels of B-type natriuretic peptide and its N-terminal fragment in heart failure: a systematic review and meta-analysis
Source: Eur Geriatr Med. 2024 Mar 8;15(4):893–901. doi: 10.1007/s41999-024-00950-x (PMC11377361; doi:10.1007/s41999-024-00950-x)
Supplement: Supplementary file 14 — Supplementary file14 (DOCX 15 KB) [file 41999_2024_950_MOESM14_ESM.docx]

| Study | D1 | D2 | D3 | D4 | D5 | D6 | D7 | D8 |
| --- | --- | --- | --- | --- | --- | --- | --- | --- |
| Nishio  2023 | Low risk | Low risk | Low risk | Some concerns | Low risk | Low risk | Low risk | Low risk |
| Katano  2022 | Low risk | Low risk | Low risk | Some concerns | Low risk | Low risk | Low risk | Low risk |
| Sato  2020 | Low risk | Low risk | Low risk | Some concerns | Low risk | Low risk | Low risk | Low risk |
| von Haehling  2020 | Low risk | Low risk | Low risk | Some concerns | Low risk | Low risk | Low risk | Low risk |
| Tsuji  2019 | Low risk | Low risk | Low risk | Some concerns | Low risk | Low risk | Low risk | Low risk |
| Thomas  2018 | Low risk | Low risk | Low risk | Some concerns | Low risk | Low risk | Some concerns | Some concers |
| Tschuida  2018 | Low risk | Low risk | Low risk | Some concerns | Low risk | Low risk | Some concerns | Some concerns |

**Table S5.** Quality assessment of the six included cohort-based observational studies exploring the impact of ASM using the Cochrane RoB tool.

D1: Selection of exposed vs. non-exposed cohorts drawn from same population; D2: Confidence in assessment of exposure; D3: Confident in outcome of interest not at start of study; D4: Matching exposed and unexposed for all variables associated with outcome of interest; D5: Confident in assessment of presence/absence of prognostic factors; D6: Confident in assessment of outcome; D7: Adequate follow-up of cohorts; D8: Co-intervention similar between groups?
